# Severe community-acquired pneumonia: timely management measures in the first 24 hours

**DOI:** 10.1186/s13054-016-1414-2

**Published:** 2016-08-28

**Authors:** Jason Phua, Nathan C. Dean, Qi Guo, Win Sen Kuan, Hui Fang Lim, Tow Keang Lim

**Affiliations:** 1 Division of Respiratory and Critical Care Medicine, University Medicine Cluster, National University Hospital, National University Health System, Tower Block, Level 10, 1E Kent Ridge Road, Singapore, 119228 Singapore; 2 Department of Medicine, Yong Loo Lin School of Medicine, National University of Singapore, Singapore, Singapore; 3 Department of Medicine, University of Utah School of Medicine, Salt Lake City, UT USA; 4 Division of Pulmonary and Critical Care Medicine, Department of Medicine, Intermountain Medical Center, Salt Lake City, UT USA; 5 Department of Respiratory Medicine, Affiliated Futian Hospital, Guangdong Medical College, Shenzhen, Guangdong China; 6 Guangzhou Institute of Respiratory Diseases (State Key Laboratory of Respiratory Diseases), First Affiliated Hospital, Guangzhou Medical University, Guangzhou, Guangdong China; 7 Department of Emergency Medicine, National University Hospital, National University Health System, Singapore, Singapore; 8 Department of Surgery, Yong Loo Lin School of Medicine, National University of Singapore, Singapore, Singapore

**Keywords:** Sepsis, Care bundles, Resuscitation, Emergency department, Intensive care unit

## Abstract

Mortality rates for severe community-acquired pneumonia (CAP) range from 17 to 48 % in published studies.

In this review, we searched PubMed for relevant papers published between 1981 and June 2016 and relevant files. We explored how early and aggressive management measures, implemented within 24 hours of recognition of severe CAP and carried out both in the emergency department and in the ICU, decrease mortality in severe CAP.

These measures begin with the use of severity assessment tools and the application of care bundles via clinical decision support tools. The bundles include early guideline-concordant antibiotics including macrolides, early haemodynamic support (lactate measurement, intravenous fluids, and vasopressors), and early respiratory support (high-flow nasal cannulae, lung-protective ventilation, prone positioning, and neuromuscular blockade for acute respiratory distress syndrome).

While the proposed interventions appear straightforward, multiple barriers to their implementation exist. To successfully decrease mortality for severe CAP, early and close collaboration between emergency medicine and respiratory and critical care medicine teams is required. We propose a workflow incorporating these interventions.

## Background

Community-acquired pneumonia (CAP) has plagued humankind for millennia. Hippocrates described pneumonia as a disease which the “ancients” named, and stated that “when pneumonia is at its height, the case is beyond remedy if he is not purged” [1]. Prognosis remained bleak through the centuries. Osler, widely recognised as the father of modern medicine, called pneumonia the “captain of the men of death” in 1901 [2]. Although outcomes improved with the advent of antibiotics, CAP continues to be one of the world’s leading causes of hospitalisation, morbidity, and mortality [3, 4]. Studies have shown that between 2 and 24 % of patients presenting to hospitals with CAP require admission to an ICU [5–7]. Hospital mortality rates for these patients range from 17 to 49 % in large multicentre cohort studies [8–10], and there are conflicting data on whether these rates are increasing or decreasing over time [11, 12].

Calls have been made to treat CAP as an emergency, with aggressive interventions to lower mortality [13]. Unfortunately, while national and international clinical practice guidelines for CAP typically review severity assessment, diagnostic tools, and choice of antibiotics, they do not emphasise the importance of timely resuscitation and care of respiratory failure [14–17]. In this review, we will explore how early and aggressive management measures may result in decreased mortality in severe CAP. We focus on the impact of management measures implemented within the first 24 hours and carried out both in the emergency department (ED) and in the ICU. These measures comprise those which specifically target severe CAP, such as identification and antibiotics, as well as those which target its complications, including septic shock and respiratory failure.

## Methods

We electronically searched PubMed (1981–June 2016) using a sensitive search strategy without language restrictions and with the following Medical Subject Headings (MeSH®) terms: “pneumonia”, “mortality”, “severe”, “severity assessment tools”, “severity scores”, “emergency service, hospital”, and “intensive care units”, “antibiotics”, “sepsis”, “shock septic”, “resuscitation”, “early goal-directed therapy”, “hypoxaemia”, “acute respiratory distress syndrome”, “patient care bundles”, and “quality improvement”. We supplemented the search by reviewing references of included studies and our files. The findings of key papers on the impact of various management measures on mortality are summarised in Table 1. For key studies on sepsis and respiratory failure which also enrolled patients without severe CAP, we recorded the proportion of patients with pneumonia. We used the principles of the Grading of Recommendations Assessment, Development and Evaluation (GRADE) system to assess the quality of evidence as high (well-done randomised trials), moderate (downgraded randomised trials or upgraded observational studies), low (well-done observational studies), or very low (downgraded observational studies) [18].

**Table 1:** Early management measures and impact on mortality in severe community-acquired pneumonia and its complications

Intervention	Patient population	Number of patients	Number with pneumonia^a^	Mortality reduction	Mortality definition	Risk (95 % CI)	Evidence quality	Selected reference
CAP severity assessment tools to guide management	Severe CAP in hospital	348	348 with CAP	Yes	Hospital	Adjusted OR 0.24 (0.09–0.67)	Low; before-and-after study	[27]
Guideline-concordant antibiotics	CAP in hospital	1288	1288 with CAP	Yes	Hospital mortality	Adjusted OR 0.55 (0.30–0.90)	Low; observational study	[39]
Macrolide combination treatment	Severe CAP in ICUs	8872	8872 with CAP	Yes	Hospital, ICU, 28-day or 30-day	RR 0.84 (0.71–1.00)	Moderate; systematic review of observational studies	[48]
Early antibiotics within 1 hour	Sepsis and septic shock	11,017	Data not available	Uncertain	Hospital or 28-day	OR 0.68 (0.42–1.12)	Moderate; systematic review of observational studies	[56]
Early antibiotics after septic shock	Septic shock	2154	838	Yes	Hospital	Adjusted OR 0.89 (0.88–0.91) per hour earlier	Low; observational study	[55]
Early goal-directed therapy	Septic shock	4201	1278	Uncertain	90-day	OR 0.99 (0.86–1.15)	High; systematic review of RCTs	[66]
High-flow nasal cannula	PaO_2_/FIO_2_ ≤ 300 mmHg	310	254 (197 with CAP)	Yes	90-day	HR 0.50 (0.25–0.99)	High; RCT	[71]
Low tidal volumes and plateau pressures	ARDS with PaO_2_/FIO_2_ ≤ 300 mmHg	861	69	Yes	Hospital	RR 0.78 (0.65–0.93)	High; RCT	[76]
High positive-end expiratory pressure	ARDS with PaO_2_/FIO_2_ ≤ 200 mmHg	2299	1145	Yes	Hospital	Adjusted RR 0.90 (0.81–1.00)	Moderate; systematic review of RCTs; conflicting results with other systematic reviews	[79]
Low driving pressure	ARDS with PaO_2_/FIO_2_ ≤ 300 mmHg	3562	1314^b^	Yes	Hospital at 60 days	RR 0.71 (0.66–0.76) for each 1-SD decrease in driving pressure	Moderate; systematic review of RCTs of different objectives and methods	[77]
Neuromuscular blockade	ARDS with PaO_2_/FIO_2_ < 150 mmHg	339	262 (130 with CAP)	Yes	90-day	Adjusted HR 0.68 (0.48–0.98)	High; RCT	[81]
Prone positioning	ARDS with PaO_2_/FIO_2_ < 150 mmHg	466	281	Yes	28-day	HR 0.39 (0.25–0.63)	High; RCT	[83]
Corticosteroids	Severe CAP according to severity assessment tools	388	388 with CAP	Uncertain	Varies between RCTs	RR 0.39 (0.20–0.77)	Moderate; systematic review of RCTs; conflicting results with other systematic reviews	[87]
Care bundles	CAP in hospital	2118	2118 with CAP	Yes	30-day	Adjusted OR 0.59 (0.37–0.95)	Low; before-and-after study	[97]

^a^Numbers include all forms of pneumonia, including CAP and hospital-acquired pneumonia. Number of CAP patients is stated where available

^b^A total of 1314 out of 3449 patients had pneumonia (specific data on pneumonia were not available from one study of 113 patients [106])

*CI* confidence interval, *OR* odds ratio, *RCT* randomised controlled trial, *PaO*_*2*_ partial pressure of arterial oxygen, *FIO*_*2*_ fraction of inspired oxygen, *HR* hazard ratio, *ARDS* acute respiratory distress syndrome, *RR* risk ratio, *SD* standard deviation, *CAP* community-acquired pneumonia

### Early recognition of severe CAP

Severity assessment tools may help clinicians recognise severe CAP and select patients for early intervention. In 1997, Fine et al. [19] showed that the Pneumonia Severity Index (PSI) predicts mortality. This was followed by validation of the CURB65 (Confusion, Urea, Respiratory Rate, Blood pressure, Age > 65 years) score [20]. However, while both the PSI and the CURB65 score guide decisions to hospitalise patients, they were not designed to guide ICU admission. After two earlier iterations [21, 22], the American Thoracic Society (ATS) and the Infectious Diseases Society of America (IDSA) recommended a set of major and minor criteria for ICU admission in 2007 [14]. The major criteria are invasive mechanical ventilation and/or the need for vasopressors. Fulfilment of the minor criteria requires three or more of the following: tachypnoea, hypoxaemia, multilobar infiltrates, confusion, uraemia, leukopaenia, thrombocytopaenia, hypothermia, and hypotension. Several groups have validated these criteria using existing pneumonia databases [23–26].

Two systematic reviews and meta-analyses found 40 studies on different severity assessment tools to guide ICU admission [5, 6]. Marti et al. [5] found that the 2007 IDSA/ATS rule had a pooled sensitivity and specificity of 84 % and 78 % respectively for the prediction of ICU admission, while Chalmers et al. [6] found a pooled sensitivity and specificity of 61 % and 89 % respectively. When the major criteria were removed from the rule, the minor criteria had a pooled sensitivity and specificity of 57 % and 90 % respectively according to Marti et al. [5], and of 56 % and 92 % respectively according to Chalmers et al. [6]. Other similar tools include the SMART-COP (Systolic blood pressure, Multilobar infiltrates, Albumin, Respiratory rate, Tachycardia, Confusion, low Oxygen, low PH) which had a pooled sensitivity and specificity of 79 % and 64 % respectively, and the SCAP (Severe Community-Acquired Pneumonia) score which had a pooled sensitivity and specificity of 94 % and 46 % respectively [5].

Severity assessment tools have several limitations [13]. Firstly, they do not differentiate preventable mortality (which may be reduced by timely management measures) and non-preventable mortality (such as in older patients with multiple co-morbidities and do-not-resuscitate orders), and evidence that adoption of these tools may improve outcomes remains weak [27]. This concern is partly mitigated by tools such as the IDSA/ATS minor criteria, which do not contain age or co-morbid illness factors and therefore select patients based on acute physiology, as opposed to tools such as the PSI [14, 19]. Secondly, studies of severity assessment tools often did not exclude patients with orders to withhold life-sustaining treatments, thus compromising the validity of their findings [5, 6]. Thirdly, because their sensitivity and specificity are not 100 %, some patients who would have done well even without close monitoring may be admitted to an already resource-limited ICU, while others at risk for death would not be detected [5, 6]. Fourthly, decisions for ICU admission are dependent on local culture and resources [28]. Some groups have used the need for mechanical ventilation and vasoactive agents rather than ICU admission per se as end points [29–31]. Fifthly, it remains to be seen how these tools which are specifically for CAP compare with more generic criteria for sepsis such as the recently introduced quick Sequential Organ Failure Assessment (qSOFA) score, which predicts poor outcomes in the presence of at least two of the following: systolic blood pressure ≤ 100 mmHg, respiratory rate ≥ 22/min, or altered mentation [32].

### Early action with or without ICU admission

Much work has been devoted to finding the ideal severity assessment tool to guide ICU admission because several studies have linked delayed ICU admission for severe CAP with increased mortality [33–36]. Such a finding, however, does not necessarily mean that earlier ICU admissions will result in improved survival. Some patients are admitted late to the ICU because of subsequent deterioration and not because they are unwell to begin with [37]. This notwithstanding, the adverse effect on mortality of admitting patients from an ED to a general ward rather than straight to an ICU persisted even when accounting for late deteriorations by adding radiographic progression to a logistic regression model [38]. Other patients are admitted to the ICU late because clinicians may sometimes inadvertently underestimate the severity of illness in the ED [38]. In these cases, earlier ICU admission alone is insufficient, and must be paired with aggressive management measures in the ED. Indeed, patients in a Singaporean study deemed critically ill and directly transferred from the ED to the ICU received more fluids and antibiotics in the ED than those triaged to the general wards and transferred to the ICU later [35].

Given the limitations of severity assessment tools, we argue that it is time to move on from the search for the perfect tool to effective translation of currently available tools into action. Lim et al. [27] linked CAP severity assessment tools to management prior to initiation of mechanical ventilation or vasopressors. Their multifaceted intervention used the 2007 IDSA/ATS minor criteria to identify severe CAP early in the ED and to trigger empiric antibiotics within 3 hours of triage, prompt intubation for respiratory failure, fluid resuscitation, and vasopressors for patients with hypotension or shock. This before-and-after study reported a decrease in the hospital mortality rate from 24 % to 6 % (Table 1). While inappropriately delayed ICU admissions decreased from 32 % to 15 %, the intervention also decreased the overall ICU admission rates from 53 % to 39 % because early and appropriate resuscitative measures in the ED helped to stabilise patients who could then be managed on a general ward.

### Early antimicrobial therapy

Common causative organisms for severe CAP include *Streptococcus pneumoniae*, *Staphylococcus aureus*, *Legionella* species, Gram-negative bacilli, *Haemophilus influenzae*, and influenza A and B viruses [14, 15, 17]. Adherence to guidelines for empiric antibiotics is associated with improved survival [39, 40] (Table 1), although microbial aetiology varies across time and place, and different guidelines have proposed slightly different antimicrobial regimes. In general, however, American, British, and European guidelines all recommend empirically starting a beta-lactam (such as amoxicillin-clavulanate, ampicillin-sulbactam, cefotaxime, or ceftriaxone) plus a macrolide (such as azithromycin or clarithromycin) [14–17]. American and European guidelines suggest that a fluoroquinolone (such as levofloxacin or moxifloxacin) may be substituted for the macrolide [14, 17].

Several systematic reviews which explored the role of macrolides in CAP have arrived at different conclusions, depending on the type of studies included. In general, lower-quality observational studies tend to suggest survival benefits [41, 42], as opposed to studies on non-critically ill patients and randomised trials [43, 44]. Two high-quality randomised trials were recently published: a Dutch trial concluded that beta-lactam monotherapy was non-inferior to beta-lactam plus macrolide combination for 90-day mortality [45], but a Swiss trial could not demonstrate non-inferiority [46]. While these trials included non-ICU patients, macrolides have immune-modulatory effects in addition to antimicrobial properties which may benefit sicker and more septic patients [47]. Indeed, in a systematic review of 25 observational studies of 8872 patients with severe CAP, combination treatment with macrolides was associated with lower mortality compared with treatment without macrolides, including when guideline-concordant regimens of beta-lactam/macrolide and beta-lactam/fluoroquinolone were specifically compared [48] (Table 1).

Multidrug resistance is increasingly of concern, and American and European guidelines also suggest that when a *Pseudomonas* infection is suspected, the beta-lactam used should have antipseudomonal effects (such as piperacillin-tazobactam, cefepime, imipenem, or meropenem) [14, 17]. In addition, the IDSA/ATS recommended in 2005 [49] that extended-spectrum antibiotics should be used for patients admitted from the community if they have risk factors for harbouring resistant organisms, such as a recent hospitalisation, residence in a nursing home or extended care facility, home infusion therapy, chronic dialysis, home wound care, or a family member with a multidrug-resistant pathogen. This form of pneumonia was termed healthcare-associated pneumonia (HCAP). Recent work, however, has shown that the HCAP criteria poorly predict the presence of resistant organisms, and that administration of broad-spectrum antibiotics based on these criteria does not improve outcomes [50–52].

A recent systematic review concluded from four large observational studies that administration of antibiotics for CAP within 4–8 hours of hospital arrival was associated with a 5–43 % relative reduction in mortality, even in non-ICU patients [41]. Multiple investigators have associated early administration of appropriate antibiotics with improved survival in sepsis [53, 54]. Kumar et al. [55] reported that each hour of delay in antibiotic administration lowered survival by 8 % in septic shock (838 out of 2154 patients had pneumonia) (Table 1). However, a recent systematic review of 11 studies which reported the time from recognition of sepsis or septic shock to antibiotic administration did not find such an association [56] (Table 1). The latter analysis, however, must be interpreted with caution due to selection bias from excluded studies, the lack of microbiological data, and the lack of confidence that all patients studied had bacterial sepsis [57]. The Surviving Sepsis Campaign recommends administering antibiotics within the first hour of recognition of sepsis and septic shock, and the same is prudent for severe CAP [54, 58].

### Early haemodynamic support

Pneumonia is the most common cause of sepsis and often presents with septic shock [59, 60]. In a recent multicentre Spanish study, one-third of hospitalisations for CAP were complicated by sepsis [61]. The Third International Consensus Definitions for Sepsis and Septic Shock (Sepsis-3) [32] state that patients with septic shock can be identified clinically by a vasopressor requirement to maintain a mean arterial pressure ≥ 65 mmHg, and a serum lactate level ≥ 2 mmol/L after adequate fluid resuscitation, although some controversy exists because most studies from which these criteria were derived measured lactate upon presentation and before fluids. In 2001, Rivers et al. [62] showed that a protocol for haemodynamic optimisation known as early goal-directed therapy (EGDT) decreased hospital mortality in sepsis and septic shock from 46.5 % to 30.5 %. The EGDT bundle included lactate measurement, fluid resuscitation according to the central venous pressure, vasoactive agents to keep the mean arterial pressure at 65–90 mmHg, and red blood cell transfusion and/or inotropes according to the central venous oxygen saturation. Pneumonia accounted for 39 % of the enrolled patients and was the commonest cause of sepsis in the study.

In 2014 and 2015, however, three large multicentre randomised trials—the ProCESS, ARISE, and ProMISe studies [63–66]—reported that EGDT was not superior to usual care for ED patients with septic shock (Table 1). While these studies examined sepsis in general, the findings are probably applicable to pneumonia which was the leading source of infection, affecting 1278 out of the enrolled 4201 patients. Subgroup analysis by site of infection in the ProCESS study did not reveal significant differences when compared with the full analysis [63]. It is likely that the data from Rivers et al. [62] pushed clinicians towards more aggressive and early resuscitation in recent years, such that the majority of patients in the usual care arms of the ProCESS, ARISE, and ProMISe studies received prompt fluids and vasopressors, even though central venous pressure and central venous oxygenation were not targeted [63–68].

### Early respiratory support

Acute respiratory failure frequently complicates severe CAP; the combined impact of acute respiratory failure and sepsis on mortality is exponential [69]. Delay in oxygenation assessment using pulse oximetry or arterial blood gas measurements beyond 3 hours from the time of triage at hospital admission is independently associated with increased mortality [70].

In the multicentre FLORALI study, 310 patients with acute hypoxaemic respiratory failure and a ratio of the partial pressure of arterial oxygen to the fraction of inspired oxygen (PaO_2_/FIO_2_) ≤ 300 mmHg, but without haemodynamic instability, were randomised within 3 hours of meeting inclusion criteria to high-flow oxygen therapy through a nasal cannula, standard oxygen therapy delivered through a face mask, or non-invasive ventilation [71]. There were 197 patients with CAP, 37 patients with hospital-acquired pneumonia, and 20 patients with pneumonia and an immunocompromising condition. The assigned treatments were commenced a median time of 60 minutes after randomisation. Although high-flow nasal cannulae did not reduce intubation rates, ventilator-free days were increased at day 28 and survival was improved at day 90 (Table 1).

However, use of high-flow nasal cannulae and non-invasive ventilation must not delay endotracheal intubation when needed. Delay in intubation beyond 3 days from the onset of CAP symptoms has been associated with increased mortality [72]. Among patients in a French study who were intubated after failure of non-invasive ventilation for severe CAP, those with a longer delay in intubation were more likely to die [73]. A Korean study showed that delay in intubation after a failed trial of high-flow nasal cannulae increased mortality [74]. Criteria to prompt clinicians to intubate may help prevent such delays. For example, the FLORALI study advised intubation in the presence of haemodynamic instability, neurological deterioration, or persisting or worsening respiratory failure, as defined by two or more of the following: respiratory rate > 40 breaths per minute, signs of high respiratory-muscle workload, copious tracheal secretions, arterial pH < 7.35, and pulse oximetry reading < 90 % for >5 minutes [71].

Pneumonia is the most common cause of acute respiratory distress syndrome (ARDS) [75], and as detailed in Table 1 accounts for a large proportion of participants in multiple ARDS trials. Lung-protective ventilation with low tidal volumes of 6 ml/kg of predicted body weight and limitation of the driving pressure (tidal volume divided by respiratory system compliance) after intubation have been associated with reduced mortality [76, 77] (Table 1). The impact of limiting tidal volumes on mortality is greatest at the start of mechanical ventilation [78]. A patient-level meta-analysis of three large multicentre randomised trials suggested that higher positive-end expiratory pressure may improve survival [79], although subsequent systematic reviews have not found a similar association [80] (Table 1). Early initiation of a 48-hour infusion of cisatracurium for neuromuscular blockade for patients with severe hypoxaemia (PaO_2_/FIO_2_ < 150 mmHg) lowers mortality [81, 82], as does early prone positioning [83, 84] (Table 1).

### Early corticosteroids

While systemic corticosteroids attenuate the inflammatory response in CAP, recent data on their role are conflicting. In a multicentre randomised controlled trial of 785 patients, 386 of whom had severe CAP as defined by PSI classes IV and V, Blum et al. [85] found that corticosteroids shortened the time to clinical stability. In another trial of 120 patients with severe CAP and C-reactive protein levels > 150 mg/L, Torres et al. [86] found that corticosteroids decreased treatment failure, although predominantly by halting radiographic progression rather than improving patient-centric outcomes. Three recent meta-analyses came to different conclusions, with two analyses suggesting that corticosteroids decrease mortality in severe CAP [87, 88] (Table 1) and one analysis finding no impact on mortality [89]. The latter review, however, suggested that corticosteroids are safe, and may reduce the risk of ARDS, lengths of hospital and ICU stay, and time to clinical stability. These meta-analyses are limited by the heterogeneity of the included studies, and in particular are skewed by an outlying randomised trial by Confalonieri et al. [90] which found that hydrocortisone reduced mortality from 38 % to 0 %. More data are thus needed before corticosteroids become routine treatments for severe CAP [91].

### Early use of care bundles

Care bundles combine several management practices to improve outcomes [53]. In 2002, the Surviving Sepsis Campaign recommended that a resuscitation bundle should be performed within 6 hours for septic patients [92]. The bundle included principles from EGDT, in addition to blood cultures and broad-spectrum antibiotics. Recent international analyses by the Surviving Sepsis Campaign showed that compliance to the guidelines was associated with decreased mortality [58, 93].

A few before-and-after studies have focused on sepsis care bundles derived from the Surviving Sepsis Campaign guidelines [58] for severe CAP. In a single-centre study in China, Guo et al. [94] defined severe CAP according to the IDSA/ATS major criteria and applied the Surviving Sepsis Campaign’s 6-hour resuscitation bundle (and a 24-hour management bundle) for these patients. While the intervention was associated with a decrease in overall hospital mortality from 44 % to 29 %, full compliance to the bundles was associated with a greater than twofold decrease. Georges et al. [95] reported that a similar bundle including antibiotic therapy, fluids, and vasoactive agents was associated with a decrease in mortality from 43 % to 31 % in patients with severe CAP as defined by ICU admission in a single French centre. Hortmann et al. [96] implemented a care bundle for all patients with CAP in a German ED. The bundle comprised checklists on history, clinical examination, investigations including lactate measurement and blood cultures, risk stratification according to CRB65, and treatment including antibiotic guidelines and fluid resuscitation. Hospital mortality decreased from 14 % to 11 %. Sixteen United Kingdom hospital trusts participated in a quality improvement programme incorporating a British Thoracic Society care bundle which included the use of the CURB65 score, standardised oxygen assessment and prescription, and chest X-ray scan and targeted antibiotics within 4 hours of hospital admission [97]. Bundle implementation was associated with improved 30-day inpatient mortality (Table 1). Dean et al. [98] showed that four hospital EDs experienced lower CAP mortality after introduction of an electronic clinical decision support tool compared with three usual care hospitals. The tool had multiple features, including electronic calculation of the IDSA/ATS minor criteria coupled with logic for ICU admission. Treatment protocols in the EDs and ICUs guided management [99].

### Early collaboration between teams

Without explicit guidelines, clinical management differs between individual physicians [100]. Workflows that incorporate the interventions reviewed here are needed, but multiple barriers slow their adoption. Using the framework drawn by Cabana et al. [101], these barriers include: a lack of awareness of, familiarity with, or agreement with the workflows, lack of self-efficacy (e.g. confidence in inserting central venous catheters for vasopressors), disbelief that the workflows can reduce mortality, inertia, and external barriers such as cumbersome workflows, insufficient staff, and insufficient time. Overcoming these barriers requires close liaison between emergency medicine, respiratory medicine, and critical care medicine clinicians, as well as clinical decision support tools. These are unfortunately not emphasised in current guidelines for CAP and sepsis [14–17].

To illustrate, Lim et al.’s intervention [27] was designed and sustained by the same multidisciplinary team over 7 years, comprising representatives from the ED and the respiratory and critical care medicine department. Local champions trained nurses and physicians on the definitions and management of severe CAP. Orientation tutorials were provided for new staff, and posters and forms were displayed prominently. Data on compliance were reviewed every 2–4 weeks during business meetings and email discussions, and regular feedback was obtained to improve the workflow. These processes continue to this day. Guo et al. [94] used a personal pocket information card as a daily reminder of the steps required for severe CAP, and provided weekly feedback to staff on compliance to their workflow through group discussions and posters. Dean et al. [98] not only deployed their electronic pneumonia clinical decision support tool, but also closely followed the principles of change management.

### The first 24 hours

**Fig. 1:**
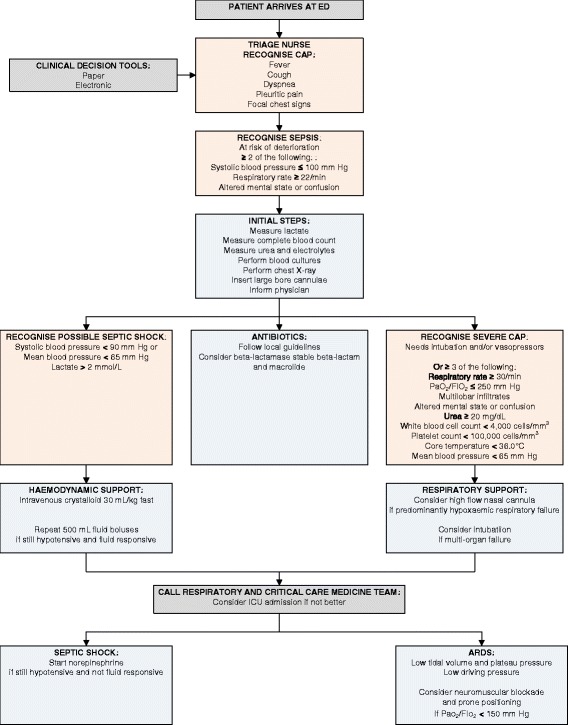
Suggested approach to early and aggressive management measures for severe community-acquired pneumonia (*CAP*). *ED* emergency department, *PaO*_*2*_ partial pressure of arterial oxygen, *FIO*_*2*_ fraction of inspired oxygen

We propose in Fig. 1 a bundled approach to early and aggressive treatment to improve survival for patients with severe CAP. The algorithm is guided by clinical decision support tools available in the ED in paper or electronic form [27, 94, 98]. The approach relies on ED nurses to rapidly diagnose CAP at triage, and then suspect sepsis using the qSOFA score by identifying low blood pressure, tachypnoea, and altered mental state [32]. It empowers the nurses to order lactate measurements, blood cultures, and chest X-ray scans, to insert large-bore intravenous cannulae, and to alert the emergency physicians [27]. Broad-spectrum antibiotics must be given early, and include a beta-lactamase-stable beta-lactam and a macrolide [14–17, 39, 48, 56]. Patients with hypotension and/or elevated lactate are given boluses of crystalloids [32, 58]. Severe CAP is defined by the need for intubation and/or vasopressors, or by more subtle features such as the IDSA/ATS minor criteria [14]. High-flow nasal cannulae may be considered for respiratory failure [71], but the application of stringent semi-elective intubation criteria is preferred to emergent intubation near the point of cardiorespiratory arrest [71–73]. The respiratory and critical care medicine department(s) should be engaged early. Patients who do not improve with initial management measures should be managed in the ICU [27, 98, 102], where norepinephrine may be infused for septic shock [103] and lung-protective ventilation with low tidal volumes and driving pressures is provided for ARDS. Prone positioning and/or neuromuscular blockade may be needed for moderate to severe cases [76, 77, 79, 81, 83].

The proposed approach has its limitations and must be adapted to each individual setting. Selected antimicrobials should be tailored to the local antibiogram. While we have focused on antibiotics for bacterial pathogens, empiric treatment with neuraminidase inhibitors for influenza in the presence of typical symptoms should be considered [14, 104]. Resource-limited settings may not be able to measure lactate levels, and more work is needed to evaluate the clinical utility of the qSOFA score [32]. The IDSA/ATS minor criteria are featured because they are well recognised, validated, and easy to use, but no severity assessment tool has perfect sensitivity or specificity [5, 6, 105] so the physicians’ clinical judgment remains key. Importantly, the workflow is highly dependent on a seamless working relationship between nurses and doctors, and between ED and respiratory and critical care medicine teams.

## Conclusions

Severe CAP has claimed too many lives for too long. The emergency medicine and respiratory and critical care medicine communities should work together to decrease mortality by implementing early and aggressive management measures upon recognition of severe CAP.

## Abbreviations

ARDS, acute respiratory distress syndrome; ATS, American Thoracic Society; CAP, community-acquired pneumonia; CI, confidence interval; CURB65, Confusion, Urea, Respiratory Rate, Blood pressure, Age > 65 years; ED, emergency department; EGDT, early goal-directed therapy; FIO_2_, fraction of inspired oxygen; GRADE, Grading of Recommendations Assessment, Development and Evaluation; HCAP, healthcare-associated pneumonia; IDSA, Infectious Diseases Society of America; MeSH®, Medical Subject Headings; OR, odds ratio; PaO_2_, partial pressure of arterial oxygen; PSI, Pneumonia Severity Index; qSOFA, quick Sequential Organ Failure Assessment; SCAP, Severe Community-Acquired Pneumonia; SMART-COP, Systolic blood pressure, Multilobar infiltrates, Albumin, Respiratory rate, Tachycardia, Confusion, low Oxygen, low PH
